# Drug-Induced Autoimmune Hepatitis: Robust Causality Assessment Using Two Different Validated and Scoring Diagnostic Algorithms

**DOI:** 10.3390/diagnostics15131588

**Published:** 2025-06-23

**Authors:** Rolf Teschke, Axel Eickhoff, Gaby Danan

**Affiliations:** 1 Department of Internal Medicine II, Division of Gastroenterology and Hepatology, Klinikum Hanau, D-63450 Hanau, Germany; eickhoff.axel@gmx.de; 2 Academic Teaching Hospital of the Medical Faculty, Goethe University Frankfurt/Main, D-60629 Frankfurt am Main, Germany; 3 Pharmacovigilance Consultancy, Rue des Ormeaux, 75020 Paris, France; gaby.danan@gmail.com

**Keywords:** DIAIH, idiosyncratic DILI, autoimmune hepatitis, updated RUCAM, simplified AIH score

## Abstract

Drug-induced autoimmune hepatitis (DIAIH) is a relatively new subtype of idiosyncratic drug-induced liver injury (iDILI), but the features of DIAIH have been variably described due to the inhomogeneity of assessed study cohorts. The aim of this analysis is to harmonize DIAIH cohorts by unifying causality assessments, which may help characterize the features of DIAIH. Methods: Published reports of DIAIH cases were evaluated for the causality assessment methods used to verify the diagnosis of DIAIH. This disorder consists of two parts, i.e., the iDILI part and the autoimmune (AIH) part, whereby each part needs a specific diagnostic algorithm. The validated and scoring Roussel Uclaf Causality Assessment (RUCAM) is privileged for assessing the iDILI part, and the validated, simplified AIH score is the perfect choice for evaluating the AIH part. The analysis of DIAIH publications revealed that 12/20 reports (60%) presented cases assessed by both the RUCAM and the simplified AIH score, providing 49 drugs and drug combinations as causative drugs in up to 25 cases of DIAIH. Serum alanine aminotransferase activities of up to 3489 UL and high titers of autoimmune parameters such as anti-nuclear antibodies, anti-smooth-muscle antibodies, and soluble liver antigen antibodies supported DIAIH diagnosis. In contrast, 4/20 reports (20%) applied only RUCAM, and 2/20 reports (10%) used only the simplified AIH score; these 6 reports therefore provided insufficient criteria for a valid DIAIH diagnosis. Moreover, 2/20 reports (10%) did not use any causality algorithm, providing elusive features of DIAIH. While DIAIH is clearly restricted to drugs as responsible agents, this term is erroneously used to refer to disease induced by non-drugs such as herbs, green tea, dimethoate (an organophosphate insecticide), dietary supplements, biologics, herbal remedies, different viruses, and bacteria, as well as vaccines. For diseases induced by these agents, a better term could be, for instance, non-drug-induced autoimmune hepatitis. Drug cessation and immunotherapy with corticosteroids and azathioprine comprise the treatment of choice. The characteristics of DIAIH can best be described if both the RUCAM and the simplified AIH score are used concomitantly.

## 1. Introduction

Idiosyncratic drug-induced liver injury (iDILI) is a multifaceted disorder that was initially characterized by cases assessed for the inauguration of the Roussel Uclaf Causality Assessment Method (RUCAM), published in 1993 [1,2]. More than 25 years later, the US LiverTox database described the RUCAM system as a means of assigning points for clinical, biochemical, serological, and radiological features of liver injury. The system gives an overall assessment score that reflects the likelihood that the hepatic injury occurred due to a specific medication [3]. The LiverTox database mentioned that the RUCAM is now widely used to assess the causality of DILI, both in the published literature and in support of regulatory decisions regarding medications implicated in causing hepatic injury. The LiverTox database reiterates the fact that the RUCAM has been evaluated for accuracy, reproducibility, and intraobserver variability and has performed moderately well. In addition, because the RUCAM score is based upon objective criteria, there should actually be little or no variation in the final scores obtained by different investigators [3]. In addition to this appreciation in the US by LiverTox, and even at the international level, the RUCAM was well evaluated in a scientometric investigation (2010–2019) incorporating knowledge mapping of iDILI [4], which provided, among other positive aspects such as top DILI researchers, a list of the top 10 co-cited references related to DILI research, with the highest ranking being attributed to the RUCAM report of 1993 [1]. In addition to the classic iDILI classification, which includes cases that do not present autoimmune features [1,2,4,5,6,7,8,9,10], the RUCAM helps characterize several iDILI subtypes, like those with genetic features of human leukocyte antigens (HLAs) [11,12,13,14], the immune system-related Stevens–Johnson syndrome, or toxic epidermal necrolysis (TEN) [15,16,17,18], as well as autoimmune findings related to cytochrome P450 (CYP) in the form of serum anti-CYP antibodies [19,20,21].

With regard to idiosyncratic drug-induced autoimmune hepatitis (DIAIH), another RUCAM-based iDILI type has attracted much clinical interest [22,23,24,25,26,27,28]. As a complex disorder, DIAIH consists of two different components, i.e., the DILI part and the autoimmune hepatitis (AIH) part, and both parts must be separately assessed regarding causality to determine whether drugs can be identified as offending agents. However, the list of causative drugs triggering DIAIH remains incomplete due to problematic causality assessments, i.e., if only one causality assessment method was used instead of two, or, even worse, if no method was used, leading to overall degradation in the quality of determinations of the agents involved in DIAIH development, from drugs as primary suspects to unverified causatives.

This review article analyzes published reports that described the clinical features of DIAIH, focusing on the issue of whether cases were characterized as having been exclusively caused by drugs or by non-drug-related agents. The RUCAM and the AIH causality scores allow for the correct description of characteristic DIAIH features secondary to drugs, and their use is described in well-validated cases. In addition, published reports on DIAIH are evaluated to determine whether the term DIAIH is being correctly applied, as in some publications, non-drug causative agents rather than drugs were implicated in DIAIH. In our review, proposals are made regarding how best to assess drug-related causality in suspected DIAIH cases.

## 2. Literature Search and Search Terms

The literature search strategy involved the PubMed database and Google Science using the following terms: DIAIH, autoimmune DILI, immune-mediated DILI, DILI with autoimmune features, DIALH, and AIH. The search was completed on 25 February 2025. Papers were preferred that used a validated causality assessment algorithm. There were no exclusion criteria, thereby allowing for broader cohort analyses.

## 3. Diagnostic Causality Assessment Algorithms

Analytically, the DIAIH consists of two components: one is the liver injury part, known as the iDILI, and the other represents the autoimmune part, as evidenced by the autoimmune markers of the mechanisms leading to the liver injury. As a consequence, two different diagnostic methods must be applied, i.e., one for the iDILI features and one for the autoimmune characteristics.

### 3.1. Roussel Uclaf Causality Assessment Method (RUCAM)

To assess drug causality in the iDILI part of suspected cases of DIAIH, the RUCAM is privileged, both in the original version [1] or better now in its update, which was published in 2016 [29]. The RUCAM benefited from early internal validation [2] as well as subsequent external validation and good interrater reliability in several reports [21,30,31]. It represents a scoring diagnostic algorithm of drugs implicated in iDILI, using key elements such as time to onset, course of liver tests after cessation of the drug use, risk factors, comedication, alternative causes, previous hepatotoxicity, and response to unintentional reexposure. Each element receives an individual score, and summing these scores gives a total score which serves as a causality grading: ≤0, excluded; 1–2, unlikely; 3–5, possible; 6–8, probable; and ≥9, highly probable [1,29]. As such, the RUCAM is considered a key diagnostic algorithm for iDILI [5].

More than 10 years after the inauguration of the original RUCAM [1,2], details on a large number of additional RUCAM-based iDILI cases were published by experts in the field [31,32]. These reports paved the emerging role of the RUCAM for worldwide application in 81,856 iDILI cases published until mid-2020 [33], outperforming thereby all alternative assessing tools listed previously [29,34] with respect to iDILI case numbers [33]. As a result, the RUCAM was used to assess the iDILI part of DIAIH cases, as published in the DIAIH reports listed below, a fact broadly ignored by as many as 35 contributors in association with the AIHG and EASL DHILI consortia, who dismissed the value of the RUCAM [35]. Instead, the contributors discussed an electronic method, a tool with as many as 11 contributors that was criticized for being unusable due to its lack of a proper internal and external validation, an obvious negligence of good clinical work by the contributors [36]. Rather than providing robust recommendations on how best to diagnose and manage patients with suspected DIAIH, the 35 contributors merely opinionized in their meeting report that current gaps prevailed with respect to the diagnosis, analysis, and management of DIAIH cases, and that this was an issue to be resolved in the future. This represented a poor outlook [35], in view of the existence of robust diagnostic algorithms like the RUCAM [1,2,29] or the validated simplified AIH score [37,38]. At odds was the game changing of positions from initially RUCAM using and promoting authors [31,32] to RUCAM dismissing contributors [35]. This counteracts the aim of diagnostic harmonization, which would be helpful if uniformity of iDILI criteria including specific scoring is to be established worldwide, allowing published data across registries to be harmonized and easily interpreted across populations [39].

### 3.2. Simplified AIH Score

To verify causality for the involvement of drugs in the autoimmune hepatitis part of DIAIH, the application of the simplified criteria of the diagnosis of the AIH, the “simplified AIH score in short”, is highly recommended. This scoring tool was published by the International Autoimmune Hepatitis Group (IAIHG) in 2008 as an internally validated diagnostic algorithm [36], with subsequent external validation in 2009 [37]. The simplified AIH score considers elements such as anti-nuclear antibodies (ANAs), liver-kidney microsomal antibodies (LKM), soluble liver antibodies (SLA), immunoglobulin G (IgG), liver histology, and the absence of viral hepatitis, while summing of the individual scores gives a final score with a specific causality grading [36]. Accordingly, a reliable diagnosis of AIH can be made using the simplified AIH score, specifying the diagnosis of a probable AIH at a cutoff score of 6 points and of a definite AIH with 7 points or higher.

The simplified AIH scoring algorithm is highly appreciated worldwide, having been quoted in 2252 publications as retrieved from Google Science. Reports have focused, among many topics, on the accuracy of AIH diagnosis in children [40], its suitability for AIH diagnosis in a German cohort [41], its comparison with primary sclerosing cholangitis [42], AIH with diagnostic and serological testing [43], its accuracy for AIH diagnoses in children, including a systematic review and decision analysis [44], a validity and efficiency evaluation in children [45], its utility in children [46], its validation and modification in children [47], its evaluation versus the revised scoring system in patients in general [48], and the histological scoring system as a significant step toward the optimization of clinical diagnoses [49]. Despite some published suggestions and validation efforts, the simplified AIH score has been found to perform very well, even in highly heterogeneous populations in different regions of the world [50].

### 3.3. Advantages of Using Both the RUCAM and Simplified AIH Score

Both the RUCAM and the simplified AIH scores are validated methods suitable to assess causality in cases of suspected DIAIH and are able to confirm or reject the diagnosis if used in combination. Indeed, they complement each other, as none of the methods alone can provide a correct diagnosis, considering that the simplified AIH score has some shortcomings regarding alternative causes like hepatitis A and E, cytomegalovirus, Epstein-Barr virus, herpes simplex virus, and other liver diseases such as Wilson disease and hemochromatosis [38]. This shortcoming can well be overcome by the RUCAM, which addresses these missing special elements as possible alternative causatives [29]. As a consequence, the present review will focus on published cases of suspected DIAIH which were carefully assessed by both the RUCAM and simplified AIH score.

## 4. Search for Causality Assessment Algorithms Applied in Suspected DIAIH

The correct diagnosis of DIAIH critically depends on the quality of the diagnostic causality assessment approach, with special focus on the RUCAM [1,29] and the simplified AIH score [38], taking as a given that the best results may be obtained if both methods are applied together. However, causality assessments of drugs as causatives in DIAIH often remain elusive when cases are incompletely assessed, i.e., using a single diagnostic algorithm—either the RUCAM or the simplified AIH score—or even worse, if no causality evaluation was done. Shortcomings in causality approaches inevitably lead to inaccurate results. For selected drugs and drug groups, various causality assessment methods were explored (Table 1) [51,52,53,54,55,56,57,58,59,60,61,62,63,64,65,66,67,68,69,70].

Although the above compilation shows that most drugs are potential culprits concerning DIAIH, and the included cases may shed some light on possible causatives responsible for DIAIH (Table 1) [51,52,53,54,55,56,57,58,59,60,61,62,63,64,65,66,67,68,69,70], several issues warrant further analysis and discussion, with a focus on the four following points: (1) While DIAIH is clearly restricted to drugs as responsible agents, this term was used erroneously in a few cohort cases involving non-drugs such as herbs [67], green tea [56], dimethoate (an organophosphate insecticide) [56], dietary supplements [71], biologics [67], herbal remedies, different viruses, and bacteria, as well as vaccines [72]. For these causatives a better term could be, for instance, non-drug-induced autoimmune hepatitis (nDIAIH); (2) Not included in Table 1 were drugs mentioned in poorly evaluated reports on immune checkpoint inhibitors (ICIs) involved in suspected DIAIH but lacking a formal causality assessment for AIH and DILI [73,74,75,76,77]; (3) Although partially of some general interest, reports lacking a list of drugs involved in DIAIH were not included [17,18,23,27,32]; (4) Other shortcomings include, for instance, if the DILI part of DIAIH was evaluated using the Naranjo method [26], a method which has not been validated for DILI because it is not specific to liver injury [29,34]; and finally, (5) A new approach using a single algorithm combining parts of a modified RUCAM with parts of a modified simplified AIH score was not considered due to missing specific drugs [22], i.e., an approach similar to the proposal to integrate the updated RUCAM in an AIH scoring system [78].

The analysis of 20 publications of DIAIH revealed that 12/20 reports (60%) presented cases assessed by both the RUCAM and the simplified AIH score [53,54,55,56,57,58,59,61,64,66,67,68]; these were appreciated by the present authors, providing refreshing and encouraging results in favor of robust DIAIH diagnoses (Table 1). In contrast, 4/20 reports (20%) applied only the RUCAM [51,60,65,70], and 2/20 reports (10%) used only the simplified AIH score [52,62], thereby providing insufficient rigor for a valid DIAIH diagnosis in these 6 reports (Table 1). Even worse, 2/20 reports (10%) did not use either of the 2 causality algorithms [63,69], providing elusive feature data concerning DIAIH. Finally, in a retrospective study, drugs were suspected to have induced AIH in 9.2% cases classified erroneously as DIAIH; this conclusion was underlined by the fact that the DILI part of these diagnoses was not established using the RUCAM (Table 1) [62].

## 5. Clinical Features of DIAIH

In this section, which is dedicated to clinical DIAIH characteristics, the data were all retrieved from DIAIH cases with diagnoses verified by both the RUCAM and the simplified AIH score, amounting to 12/20 DIAIH reports that included 49 different drugs, drug groups, and drug combinations in a total of 25 DIAIH cases (Table 1). The RUCAM scores were up to 10 [53,54], or 6–8 [57], and the AIH scores were up to 14 [53,54] or 10–17 [57].

DIAIH was often misdiagnosed if alternative causes were not correctly excluded, a problem also connected to iDILI [79]. More specifically, competing non-drug diagnoses have been detected in the course of DIAIH case evaluations [54,57,66]. As an example, a well conducted DIAIH study showed alternative causes in 35.5% of cases, on top the genuine AIH (10.5%), followed by cholangitis/cholelithiasis (5.2%), hepatitis E virus infections (4.2%), alcohol use (3.5%), cardiac failure (2.8%), secondary sclerosing cholangitis (2.1%), non-alcoholic steatohepatitis (now known as metabolic dysfunction-associated steatohepatitis) (1.7%), other autoimmune diseases (1.7%), metabolic disorders of Wilson disease and hemochromatosis (1.4%), primary biliary cholangitis (1.4%), primary sclerosing cholangitis (1.1%), non-viral infections like abscesses and echinococcus (1.1%), hepatitis A virus (0.7%), human herpes virus (0.7%), Epstein-Barr virus (0.7%), cytomegalovirus (0.4%), malignant infiltration (0.4%), and others (1.1%) [54].

Notably, the aforementioned opinion report by three dozen contributors dismissed any validated and universally used validated causality assessment method, such as the updated RUCAM and the AIH score, for evaluation of the DIAIH cases [35]. This approach was outside of any constructive scientific philosophy, because several authors have applied the RUCAM or the AIH scores in their own publications on this topic (Table 1) [54,55,64,66] or they have used RUCAM in their previous DILI papers [12,13,16,17,23,24,30,32]. Thus, the opinion report reflecting game changing issues was disappointing and should be avoided in future publications on the subject, because it lacked basic clinical and theoretical understanding, and provided no thorough approach for the diagnosis of DIAIH, comprising merely a narrative lacking evidence based DIAIH data and diagnostic criteria [35].

### 5.1. Demographic Details

The age of DIAIH patients was 56.5 years (median), with an interquartile range (IQR) of 42.7–64.7 [56], in line with similar data, showing ranges from 22 to 76 and 22 to 87 years, in two other reports [53,54]. Female gender prevailed, making up 93% [64], 91.7% [53], 63.3% [54], and 58.8% [56] of cases.

### 5.2. Duration of Causative Drug Use

The duration of drug intake was four days (median), with an IQR of 1.0–9.2 [56], but other figures concerning the latency period of 81 days (median) and a range 12–1689 days were much higher [54].

### 5.3. Symptoms and Clinical Presentation

Large cohorts focused mostly on topics outside of the symptoms and clinical presentation of individual patients experiencing DIAIH. Regarding our analysis, a better approach was therefore to search for details published in reports on individual patients. As an example, one patient experienced DIAIH following treatment with menotrophin and developed a yellowish discoloration of stool and dark urine [68]. Such unspecific symptoms are indistinguishable from other hepato-biliary disorders that may confound DIAIH diagnoses [29]. This is why the RUCAM must be used to search and exclude alternative causes [79,80]. Scleral icterus, associated with otherwise unremarkable clinical presentations, was described in another patient with DIAIH following the administration of sorafenib [58]. General jaundice was reported in another DIAIH patient observed following treatment with atorvastatin [59]. The importance of using the updated RUCAM for the exclusion of non-drug causes [79] was re-reinforced by a well conducted DIAIH study, which showed alternative causes in 35.5% of cases [54]. As a consequence, not using the RUCAM to exclude alternative causes was an issue of basic methodology in 20% of the 20 reports (Table 1) [52,54,63,69].

In another study on 28 patients with DIAIH caused by various drugs, jaundice/pruritus was observed in 20 cases, fatigue/malaise in 7 patients, abdominal pain in 3 patients, and arthralgia in 1 patient [67]. Jaundice at onset was mentioned in 8/12 patients with DIAIH [56].

### 5.4. Laboratory Data and Auto-Immune Parameters

Serum activities of alanine aminotransferase (ALT) and alkaline phosphatase (ALP) were reported rarely and with variable results. An extremely high ALT activity of 3489 U/L was described in a patient with DIAIH due to diclofenac treatment [57], while a lower ALT value was reported, i.e., 2059 U/L, in a DIAIH patient after treatment with nitrofuran [53]. In a cohort of patients with DIAIH secondary to various drugs, ALT values of up to 66.7 times the ULN were found [54], corresponding to around 2668 U/L. Serum ALP activities were mostly within the normal range or slightly increased, but at 493 U/L, moderately increased ALP activity was observed in one patient with DIAIH caused by infliximab treatment [64].

RUCAM is based on R (ratio) values defining hepatocellular injury, cholestatic injury, and mixed injury, calculated using values of ALT and ALP [29]. R values were not specifically mentioned in the DIAIH reports, but in most cases, the hepatocellular injury type prevailed. Calculation of the R value was possible from the retrospective analysis of data published on a DIAIH cohort, whereby ALT was expressed as a multiple of the upper limit of normal (ULN), with a median of 25.0 to be divided by ALP, expressed as a multiple of ULN with a median of 1.5 [56]. The division of these two parameters resulted in an R value of 16.7, which signifies hepatocellular injury as being ≥5 compared with the cholestatic injury classified as R ≤ 2, or mixed liver injury defined as 2 < R < 5 by the updated RUCAM [29]. Finally, an R value of 16.6 as a median with a range from 2.9. to 36.7 was provided in an analysis of DIAIH caused by unspecified drugs that clearly diverged from the pattern of the liver injury, i.e., hepatocellular (95.5%) compared to mixed injury (4.5%) [54].

Autoimmune parameters were among the most important diagnostic requirements to support the diagnosis of DIAIH [38]; they included ANA, anti-smooth muscle antibo-dies (ASMA), and soluble liver antigen antibodies (SLA), but often, they were not specifically mentioned in reports in reference to the suspect drug involved in DIAIH. ANA was the most frequent auto-immune parameter found, with positive titers in 77.3% of DIAIH patients [54].

To provide an overview, laboratory data and autoimmune parameters are listed as reported for a few patients with DIAIH caused by selected drugs (Table 2).

### 5.5. Liver Histology

As opposed to the RUCAM, for which a liver biopsy is not recommended [29], for the simplified AIH score, histological data are essential elements to support the diagnosis of DIAIH, as shown in 60% of published reports (Table 1). For these reasons, details concerning histological lesions associated with DIAIH were available in published reports [61,67], as follows: lobular hepatitis [61,67], multiacinar parenchymal loss with ductular reactions and inflammatory infiltrates [67], confluent necrosis [61], entrapped hepatocytes with rosette architecture [61,67], plasma cell infiltrates [67], lymphoplasmacytic infiltrates [61], features of chronic active hepatitis [67], interface hepatitis [61], portal tract expansion with inflammatory infiltrate, associated with interface hepatitis and plasma cell aggregates [67], and portal tract with aggregated eosinophiles [67].

### 5.6. Therapy Modalities and Prognosis

Patients with DIAIH commonly received—after cessation of the suspect drug—an induction therapy with immunomodulators like steroids such as prednisolone (PRED) [53] or methylprednisolone [54] combined with azathioprine (AZA) [53], while AZA alone was used as a maintenance therapy [53]. Consensus exists that the suspect drug should be withdrawn upon first suspicion of the diagnosis and that rapid improvements in liver tests are an important diagnostic criteria. The time interval until initiation of corticosteroids was reported to be 19.5 days as a mean, and as a range from 7 to 195 days [54]. The response of induction and maintenance treatment was commonly favorable [53,54,67], with acute liver failure with the need of a liver transplantation being variable among DIAIH patients, i.e., in 4.6% [54] and 14.3% [67] of cases, or not reported [53].

Death occurred in 7.1% of DIAIH patients [67]. For selected drugs, responses to cessation of the suspected causative agent or induction therapy are listed (Table 3).

## 6. Future Considerations

In the clinical context, intuition is required as the first step concerning suspected DIAIH in a patient on medication who presents with symptoms of liver injury, i.e., increased serum activities of ALT ≥ 5 times of ULN and/or ALP of ≥2 times of ULN [1,29], as well as a positive ANA titer [36]. Clinical suspicion helps focus on causality assessments via the updated RUCAM [29] and exclusion of alternative causes [29,79]. If the RUCAM-based causality for a suspect drug is probable or highly probable, then the final approach consists of a causality assessment by the simplified AIH score that comes at the end of the diagnostic program, because this requires a liver biopsy to obtain histological data [37]. New clinical approaches may help establish diagnostic immune-related biomarkers in addition to current autoimmune parameters for the AIH part; these could replace the liver histology criteria of the simplified AIH score if validated with cases of established DIAIH diagnosis. Proposals have been made to expand the autoantibody profiling that may help distinguish DIAIH from AIH; specifically, the focus was on IgG and IgM autoantibodies directed at double-stranded DNA (SCL-70, ssDNA, U1-snRPN-BB) [35]. As expected, however, none of these parameters has entered clinical practice due to a lack of evidence base and the absence of approval by validated diagnostic scoring algorithms, omissions that remain to be corrected through clinical trials. For future DIAIH studies, precautions are recommended. There must be causality assessment harmonization of the updated RUCAM, jointly with the simplified AIH score. To name cases as DIAIH, inclusions must be confined to conventional drugs, excluding all herbal drugs and dietary supplements, as well as non-drug chemicals, instead listing alternative causes that may have been recognized during the evaluation process. Such cases should be classified as non-drug-induced autoimmune hepatitis. Studies should have a prospective design, which will ensure proactive high causality gradings because of case data completeness. Preferred for publication are cases with a probable or highly probable causality grading, as assessed by both diagnostic algorithms. A list of drugs with their causality gradings, as well as liver tests, R values based on the RUCAM for liver injury pattern, autoimmune parameters, clinical data, therapy modalities, and outcome, would be a worthwhile approach in the future.

Progress is underway with a focus on immunology features in iDILI, highlighting four major idiosyncratic DILI subtypes, as verified by the RUCAM, providing a new evidence-based classification system [80,81]. Among these four subtypes are DIAIH type 1, in addition to idiosyncratic DILI type 2 (human leucocyte antigen-based idiosyncratic drug-induced autoimmune hepatitis), type 3 (termed as idiosyncratic DILI, i.e., anti-cytochrome P450-based idiosyncratic drug-induced autoimmune hepatitis) [80], and finally, idiosyncratic DILI type 4 (immune-based idiosyncratic drug-induced liver injury associated with Stevens-Johnson syndrome (SJS) and toxic epidermal necrolysis (TEN)) [80,81]. Future reports should consider these four types when presenting clinical features of iDILI cases.

## 7. Conclusions

DIAIH is a fascinating but challenging and yet poorly characterized subtype of iDILI which is worthy of further careful analysis. Characterized as a unique disorder with complex features, i.e., a DILI part and an AIH part, DIAIH will benefit from dualistic causality assessments by the updated RUCAM and the simplified AIH score, as shown in cases described in 12 excellent reports. Accordingly, our analysis revealed that overall, 49 drugs may have caused DIAIH with verified diagnoses. Among the top 10 drugs were Nitrofurantoin (*n* = 28), Infliximab (20), Minocycline (9), Atorvastatin (8), Diclofenac (6), Ibuprofen (6), Nimesulide/Ketoprofen (6), Fluvastatine (4), Metamizole (3), and AmoxicillinClavulanate/Ceftriaxone (3). A valid causality assessment was not performed in more than one third of the 20 publications with suspected DIAIH cases, providing unfounded results that remain open for discussion. DIAIH was often misdiagnosed if alternative causes were not correctly excluded, as shown in this analysis, e.g., where non-drug diagnoses as competing confounders have been detected over the course of a case evaluation. As an example, one well conducted DIAIH study showed alternative causes in 35.5% of cases, mostly concerning AIH, followed by cholangitis/cholelithiasis, hepatitis E virus, alcohol, and cardiac failure. Thus, to improve future descriptions of the features of DIAIH, additional efforts concerning case evaluations using the updated RUCAM and the simplified AIH score are required.

## Figures and Tables

**Table 1:** Selected drugs and drug groups implicated in suspected DIAIH.

Drugs and Drug Groups	Cases (*n*)	RUCAM Causality Algorithm Used	Simplified Criteria of AIH Score Used	DIAIH Verified by Both RUCAM Plus Simplified AIH Score	References
Adalimumab	1	YES	NO	NO	Ghabril, 2013 [51]
	1	NO	YES	NO	Rodrigues, 2015 [52]
	1	YES	YES	YES	Martínez-Casas, 2018 [53]
	1	YES	YES	YES	Chung, 2024 [67]
Allopurinol	1	YES	YES	YES	Chung, 2024 [67]
Amitriptyline	1	YES	YES	YES	Weber, 2019 [54]
AmoxicillinClavulanate	2	YES	YES	YES	García-Cortés, 2023 [55]
Amoxicillin + Clavulanate + Ceftriaxone	3	YES	YES	YES	Licata, 2014 [56]
Amoxicillin + Erythromycin	1	YES	YES	YES	Chung, 2024 [67]
Amoxicillin + Metronidazole	1	YES	YES	YES	Chung, 2024 [67]
Anabolic steroid	1	YES	YES	YES	Chung, 2024 [67]
Atorvastatin	2	YES	YES	YES	Yeong, 2016 [57]
	2	YES	YES	YES	Weber, 2019 [54]
	2	YES	YES	YES	García-Cortés, 2023 [55]
	1	YES	YES	YES	Tan, 2022 [58]
	1	YES	YES	YES	Tse, 2023 [59]
	1	YES	NO	NO	Khan, 2020 [60]
Candesartan	1	YES	YES	YES	Hassoun, 2023 [61]
Cephalexin	1	NO	YES	NO	Björnsson, 2010 [62]
Cephalexin + Amoxicillin	1	YES	YES	YES	Chung, 2024 [67]
Ciprofloxacin	1	YES	YES	YES	García-Cortés, 2023 [55]
	1	YES	YES	YES	Chung, 2024 [67]
Cyproterone acetate	2	YES	YES	YES	García-Cortés, 2023 [55]
Dabigatran	1	YES	YES	YES	Weber, 2019 [54]
Dexketoprofen	1	YES	YES	YES	García-Cortés, 2023 [55]
Diclofenac	1	YES	YES	YES	Yeong, 2016 [57]
	2	YES	YES	YES	Martínez-Casas, 2018 [53]
	3	YES	YES	YES	Weber, 2019 [54]
Ebrotidine	1	YES	YES	YES	García-Cortés, 2023 [55]
Efalizumab	1	YES	YES	YES	García-Cortés, 2023 [55]
Enalapril maleate	1	YES	YES	YES	Hassoun, 2023 [61]
Etanercept	2	YES	NO	NO	Ghabril, 2013 [51]
	1	YES	YES	YES	Valgeirsson, 2019 [66]
Ezetimibe	1	YES	YES	YES	García-Cortés, 2023 [55]
Fluvastatine	4	YES	YES	YES	García-Cortés, 2023 [55]
Fosfomycin	1	YES	YES	YES	Hassoun, 2023 [61]
Hydralazine	7	NO	NO	NO	de Boer, 2017 [63]
Ibandronate	1	YES	YES	YES	Hassoun, 2023 [61]
Ibuprofen	5	YES	YES	YES	Hassoun, 2023 [61]
	1	YES	YES	YES	García-Cortés, 2023 [55]
Imatinib	1	YES	YES	YES	Björnsson, 2017 [64]
	1	YES	YES	YES	Weber, 2019 [54]
	1	YES	YES	YES	Valgeirsson, 2019 [66]
Infliximab	25	YES	NO	NO	Björnsson, 2022 [65]
	8	YES	YES	YES	Björnsson, 2017 [64]
	7	YES	YES	YES	Valgeirsson, 2019 [66]
	3	NO	YES	NO	Rodrigues, 2015 [52]
	3	YES	NO	NO	Ghabril, 2013 [51]
	1	YES	YES	YES	Chung, 2024 [67]
	1	YES	YES	YES	García-Cortés, 2023 [55]
	1	YES	YES	YES	Weber, 2019 [54]
Interferon beta	1	YES	YES	YES	Weber, 2019 [54]
Irbersartan	1	YES	YES	YES	García-Cortés, 2023 [55]
Isotretionin	1	YES	YES	YES	García-Cortés, 2023 [55]
Lansoprazole	1	YES	YES	YES	Chung, 2024 [67]
Lymecycline	2	YES	YES	YES	Chung, 2024 [67]
Mefenamic acid	1	YES	YES	YES	Hassoun, 2023 [61]
Menotrophin	1	YES	YES	YES	Alqrinawi, 2019 [68]
Metamizole	3	YES	YES	YES	Weber, 2019 [54]
Methocarbamol	1	YES	YES	YES	Weber, 2019 [54]
Methyldopa	10	NO	NO	NO	de Boer, 2017 [63]
Nimesulide + Ketoprofen	6	YES	YES	YES	Licata, 2014 [56]
Minocycline	19	NO	NO	NO	de Boer, 2017 [63]
	10	NO	YES	NO	Björnsson, 2010 [62]
	4	YES	YES	YES	García-Cortés, 2023 [55]
	4	YES	YES	YES	Chung, 2024 [67]
	1	YES	YES	YES	Weber, 2019 [54]
	1	NO	NO	NO	Harmon, 2018 [69]
NSAIDs + Antibiotics	1	YES	YES	YES	Chung, 2024 [67]
Natalizumab	1	YES	YES	YES	Valgeirsson, 2019 [66]
Nitrofurantoin	24	NO	NO	NO	de Boer, 2017 [63]
	10	NO	YES	NO	Björnsson, 2010 [62]
	8	YES	YES	YES	Martínez-Casas, 2018 [53]
	7	YES	YES	YES	Chung, 2024 [67]
	5	YES	YES	YES	García-Cortés, 2023 [55]
	4	YES	YES	YES	Yeong, 2016 [57]
	3	YES	YES	YES	Björnsson, 2017 [64]
	1	YES	YES	YES	Hassoun, 2023 [61]
Olmesartan	1	YES	YES	YES	Hassoun, 2023 [61]
Orlistat	1	YES	YES	YES	García-Cortés, 2023 [55]
Pembrolizumab	1	YES	YES	YES	Weber, 2019 [54]
Pirfenidone	1	YES	NO	NO	Fortunati, 2024 [70]
Prometrium	1	NO	YES	NO	Bjornsson, 2010 [62]
Propylthiouracil	1	YES	YES	YES	Martínez-Casas, 2018 [53]
Rivaroxaban	1	YES	YES	YES	Weber, 2019 [54]
Rosuvastatin	1	YES	YES	YES	García-Cortés, 2023 [55]
Simvastatin	1	YES	YES	YES	Yeong, 2016 [57]
	1	YES	YES	YES	García-Cortés, 2023 [55]
Sorafenib	1	YES	YES	YES	Tan, 2022 [58]
Tradozone	2	YES	YES	YES	Hassoun, 2023 [61]
Valsartan	1	YES	YES	YES	Hassoun, 2023 [61]

Selected drugs implicated in DIAIH, whereby the RUCAM, either in its original version [1] or the updated version [29], was used to assess the DILI part, and the AIH part was commonly evaluated by the simplified criteria of the AIH score [39] or rarely by one of its modified versions. Abbreviations: AIH, autoimmune hepatitis; DIAIH, drug-induced autoimmune hepatitis; NSAIDs, nonsteroidal anti-inflammatory drugs; RUCAM, Roussel Uclaf Causality Assessment Method.

**Table 2:** ALT and ALP values, as well as autoimmune parameters, as described in cases of DIAIH caused by specific drugs and drug groups.

Drugs	Cases (*n*)	ALT (U/L)	ALP (U/L)	Autoimmune Parameters	References
Adalimumab	1	562	NR	ANA	Martínez-Casas, 2018 [53]
Amitriptyline	1	NR	NR	Not specified	Weber, 2019 [54]
AmoxicillinClavulanate	2	NR	NR	Not specified	García-Cortés, 2023 [55]
AmoxicillinClavulanate + Ceftriaxone	3	NR	NR	Not specified	Licata, 2014 [56]
Amoxicillin + Erythromycin	1	NR	NR	Not specified	Chung, 2024 [67]
Amoxicillin + Metronidazole	1	NR	NR	Not specified	Chung, 2024 [67]
Anabolic steroid	1	NR	NR	Not specified	Chung, 2024 [67]
Atorvastatin	2	721	NR	ANA, ASMA	Yeong, 2016 [57]
	2	NR	NR	Not specified	Weber, 2019 [54]
	2	NR	NR	Not specified	García-Cortés, 2023 [55]
	1	696	107	Unremarkable	Tan, 2022 [58]
	1	385	163	ANA	Tse, 2023 [59]
Candesartan	1	NR	NR	Not specified	Hassoun, 2023 [61]
Cefalexin + Amoxicillin	1	NR	NR	Not specified	Chung, 2024 {67]
Ciprofloxacin	1	NR	NR	Not specified	García-Cortés, 2023 [55]
Cyproterone acetate	2	NR	NR	Not specified	García-Cortés, 2023 [55]
Dabigatran	1	NR	NR	Not specified	Weber, 2019 [54]
Dexketoprofen	1	NR	NR	Not specified	García-Cortés, 2023 [55]
Diclofenac	1	3489	NR	ANA, ASMA	Yeong, 2016 [57]
	2	1491	NR	ANA, ASMA, SLA	Martínez-Casas, 2018 [53]
	3	NR	NR	Not specified	Weber, 2019 [54]
Ebrotidine	1	NR	NR	Not specified	García-Cortés, 2023 [55]
Efalizumab	1	NR	NR	Not specified	García-Cortés, 2023 [55]
Ezetimibe	1	NR	NR	Not specified	García-Cortés, 2023 [55]
Enalapril maleate	1	NR	NR	Not specified	Hassoun, 2023 [61]
Fluvastatine	4	NR	NR	Not specified	García-Cortés, 2023 [55]
Fosfomycin	1	NR	NR	Not specified	Hassoun, 2023 [61]
Ibandronate	1	NR	NR	Not specified	Hassoun, 2023 [61]
Ibuprofen	5	NR	NR	Not specified	Hassoun, 2023 [61]
	1	NR	NR	Not specified	García-Cortés, 2023 [55]
Imatinib	1	1212	205	ANA	Björnsson, 2017 [64]
	1	NR	NR	Not specified	Weber, 2019 [54]
Infliximab	10	1658	493	ANA	Björnsson, 2017 [64]
	8	NR	NR	ASMA	Valgeirsson, 2019 [66]
	1	NR	NR	Not specified	García-Cortés, 2023 [55]
	1	NR	NR	Not specified	Weber, 2019 [54]
Interferon beta	1	NR	NR	Not specified	Weber, 2019 [54]
Irbersartan	1	NR	NR	Not specified	García-Cortés, 2023 [55]
Isotretionin	1	NR	NR	Not specified	García-Cortés, 2023 [55]
Lansoprazole	1	NR	NR	Not specified	Chung, 2014 [67]
Lymecycline	2	NR	NR	Not specified	Chung, 2024 [67]
Mefenamic acid	1	NR	NR	Not specified	Hassoun, 2023 [61]
Menotrophin	1	504	366	ANA	Alqrinawi, 2019 [68]
Metamizole	3	NR	NR	Not specified	Weber, 2019 [54]
Methocarbamol	1	NR	NR	Not specified	Weber, 2019 [54]
Minocycline	4	NR	NR	Not specified	García-Cortés, 2023 [55]
	4	NR	NR	Not specified	Chung, 2024 [67]
	1	NR	NR	Not specified	Weber, 2019 [54]
Nimesulide/Ketoprofen	6	NR	NR	Not specified	Licata, 2014 [56]
Nitrofurantoin	8	2059	NR	ANA, ASMA	Martínez-Casas, 2018 [53]
	7	NR	NR	Not specified	Chung, 2024 [67]
	5	NR	NR	Not specified	García-Cortés, 2023 [55]
	4	587	NR	ANA, ASMA	Yeong, 2016 [57]
	3	1974	204	ANA	Björnsson, 2017 [64]
	1	NR	NR	Not specified	Hassoun, 2023 [61]
NSAIDs + Antibiotics	1	NR	NR	Not specified	Chung, 2024 [67]
Olmesartan	1	NR	NR	Not specified	Hassoun, 2023 [61]
Orlistat	1	NR	NR	Not specified	García-Cortés, 2023 [55]
Pembrolizumab	1	NR	NR	Not specified	Weber, 2019 [54]
Propylthiouracil	1	754	NR	ANA	Martínez-Casas, 2018 [53]
Rivaroxaban	1	NR	NR	Not specified	Weber, 2019 [54]
Rosuvastatin	1	NR	NR	Not specified	García-Cortés, 2023 [55]
Simvastatin	1	1245	NR	ANA, ASMA	Yeong, 2016 [57]
	1	NR	NR	Not specified	García-Cortés, 2023 [55]
Sorafenib	1	1004	190	Unremarkable	Tan, 2022 [58]
Tradozone	2	NR	NR	Not specified	Hassoun, 2023 [61]
Valsartan	1	NR	NR	Not specified	Hassoun, 2023 [61]

Selected drugs implicated in DIAIH, whereby the RUCAM, either in its original version [1] or the updated version [29], was used to assess the DILI part, and the AIH part was commonly evaluated by the simplified criteria of the AIH score [39] or rarely by one of its modified versions. Abbreviations: ANA, anti-nuclear antibodies; ASMA, anti-smooth muscle antibodies; DIAIH, drug-induced autoimmune hepatitis; NR, not reported; NSAIDs, nonsteroidal anti-inflammatory drugs; RUCAM, Roussel Uclaf Causality Assessment Method; SLA, soluble liver antigen antibodies.

**Table 3:** Selected drugs implicated in DIAIH with responses after cessation of the suspect drug, following therapy with unspecified immunosuppressants or after specific treatment with prednisolone and azathioprine.

Drugs	Cases (*n*)	Response of Drug Stop or Therapy	References
Adalimumab	1	CR with PRED/AZA	Martínez-Casas, 2018 [53]
	1	CR with cessation of the culprit drug	Chung, 2024 [67]
Allopurinol	1	CR with IS	Chung, 2024 [67]
Amoxicillin + Erythromycin	1	CR with IS	Chung, 2024 [67]
Amoxicillin + Metronidazole	1	CR with IS	Chung, 2024 [67]
Anabolic steroid	1	CR with IS	Chung, 2024 [67]
Atorvastatin	1	CR with PRED	Tan, 2022 [58]
	2	CR with cessation of the culprit drug	Tse, 2023 [59]
Cefalexin + Amoxicillin	1	CR with IS	Chung, 2024 [67]
Ciprofloxacin	1	CR with IS	Chung, 2024 [67]
Diclofenac	1	CR with PRED/AZA	Martínez-Casas, 2018 [53]
Diclofenac + Ibuprofen	1	IR with PRED/AZA/TAC/UCDA	Chung, 2024 [67]
Infliximab	1	CR with IS	Chung, 2024 [67]
	2	CR with cessation of the culprit drug	Chung, 2024 [67]
Lansoprazole	1	CR with IS	Chung, 2024 [67]
Menotrophin	1	CR with PRED/AZA	Agrinawi, 2019 [68]
Minocycline	1	CR with IS	Chung, 2024 [67]
NSAIDs + Antibiotics	1	CR with IS	Chung, 2024 [67]
Nitrofurantoin	8	CR with PRED/AZA	Martínez-Casas, 2018 [53]
	7	CR with IS	Chung, 2024 [67]
Propylthiouracil	1	CR with PRED/AZA	Martínez-Casas, 2018 [53]
Sorafenib	1	CR with cessation of the culprit drug	Tan, 2022 [58]

Selected drugs implicated in DIAIH, whereby the RUCAM, either in its original version [1] or the updated version [29], was used to assess the DILI part, and the AIH part was commonly evaluated by the simplified criteria of the AIH score [39] or rarely by one of its modified versions. Abbreviations: AZA, Azathioprine; CR, complete response; DIAIH, drug-induced autoimmune hepatitis; IR, incomplete response; IS, immunosuppressants (not specified further); NSAIDs, nonsteroidal anti-inflammatory drugs; PRED, Prednisolone; RUCAM, Roussel Uclaf Causality Assessment Method; TAC, Tacrolimus; UCDA, ursodeoxycholic acid.
